# Effect of Laboratory Reagents for Cleaning Modern Contamination on the *δ* ^15^N Integrity of Nitrogen‐Poor Samples

**DOI:** 10.1002/rcm.70136

**Published:** 2026-07-16

**Authors:** Kunmanee Bubphamanee, Andrew Schauer, Roger Buick

**Affiliations:** ^1^ Department of Earth & Space Sciences University of Washington Seattle Washington USA; ^2^ Virtual Planetary Laboratory NASA Nexus for Exoplanet Systems Science Seattle Washington USA

## Abstract

**Rationale:**

Nitrogen isotope values have been widely used for tracing ancient biogeochemical processes, but contaminant‐sensitive materials, such as Precambrian sedimentary rocks, meteorites, or returned extraterrestrial samples, often have low nitrogen levels and are thus vulnerable to modern organic contamination. However, the polluting effects of laboratory solvents commonly used for organic decontamination of such materials remain unquantified for nitrogen contents and isotopic ratios.

**Methods:**

Four pre‐treatment protocols using the common organic solvents dichloromethane (DCM), *n*‐hexane, and ethanol were applied to powdered quartz controls to evaluate their pollution of nitrogen concentrations and isotopic compositions. The least polluting protocol was then applied to chert samples with low nitrogen contents to assess its effectiveness in recovering primary nitrogen signatures.

**Results:**

Comparison of total nitrogen contents before and after pre‐treatments indicates that DCM introduced the most nitrogen pollution, followed by ethanol, with *n*‐hexane causing the least. DCM‐only and DCM with ethanol pre‐treatments showed a large deviation of *δ* ^15^N values compared to untreated controls, while the ethanol‐only pre‐treatment showed a positive shift in *δ* ^15^N values. Both *n*‐hexane‐only and *n*‐hexane with ethanol pre‐treatments showed consistent *δ* ^15^N values with the untreated controls. While *n*‐hexane‐only pre‐treatment had the least effect on total nitrogen content and the nitrogen isotopic composition of the untreated controls, the use of double‐solvent pre‐treatment with differing polarities is recommended to address the broad range of modern contaminants found in nature.

**Conclusions:**

The *n*‐hexane followed by ethanol pre‐treatment provided the optimal outcome for pre‐treatment and can effectively remove modern nitrogen contamination in samples with as little as 9 ppm total nitrogen without indigenous isotopic values being obscured by reagent‐derived nitrogen pollution.

## 1 Introduction

Nitrogen is one of the essential elements for biomolecules and their nitrogen isotope composition (*δ* ^15^N) in sedimentary rocks has been used to reconstruct the early biogeochemical nitrogen cycle and ancient paleoenvironments. Samples with low nitrogen contents (i.e., Precambrian rocks, meteorites, and extraterrestrial samples returned to Earth) can offer valuable information about the origin and early evolution of life but such samples are potentially prone to modern nitrogen contamination from the ubiquitous microbial biosphere and the widespread occurrence of other organic particles and volatiles in natural and laboratory settings. It is thus crucial to accurately measure the primary nitrogen isotopic and abundance signatures in such samples to interpret correctly their origin and history.

Modern organic contamination is known to pose a significant challenge in organic geochemical analyses, in particular to trace‐level elements and compounds. False positives have been reported [1, 2, 3] apparently derived from hydrocarbon‐based drilling materials [4] and multiple other sources of post‐depositional organic contamination have been identified, including surficial contamination seeping into rock matrices [5] and modern biological activity on drill core surfaces [6]. Additional contamination can occur during storage and handling, from long‐term storage in polyethylene sample bags [7], leaching of organic by‐products from plastics [8], exposure to airborne hydrocarbons in fume hoods [9], and unclean saw blades during rock cutting [4]. Such contamination can overprint the soluble organic matter fraction, particularly in materials with trace‐level organic contents. In contrast, kerogen—the insoluble organic fraction—is generally syngenetic with the host rock and less sensitive to modern contamination. In Precambrian rocks, kerogen is derived from ancient microbial biomass and its *δ* ^15^N value records the nitrogen assimilated during primary productivity, providing a crucial archive for paleoenvironmental reconstruction. However, direct kerogen extraction is analytically challenging in samples with very low total nitrogen (TN) contents (< 30 ppm). Bulk analysis represents a more practical alternative, as it preserves a proportion of the nitrogen lost from organic matter during diagenesis and metamorphism, providing a more complete nitrogen record. Therefore, removing the soluble organic fraction is critical, especially in samples with low nitrogen contents, where even small amounts of modern contamination may disproportionately affect analytical results relative to the indigenous kerogen.

To ensure the removal of modern organic contaminants, rock samples are commonly pre‐treated with organic solvents to extract soluble materials before bulk compositional analysis. However, these pre‐treatments could also introduce pollution from the solvents; in this study, pollution refers specifically to nitrogenous material introduced by organic solvents during pre‐treatment. Previous studies have documented that laboratory reagents used during sample preparation, including HCl acidification [10] and HF‐based kerogen extraction [11], can alter their nitrogen isotopic compositions (< 2‰) by selectively removing nitrogen‐bearing fractions. However, the effects of organic solvents used for sample cleaning on nitrogen concentration and isotopic composition have not been explored. To date, only one study has reported a bias in carbon concentration and isotopic composition by organic solvents used in cleaning [12]. This exploratory study addresses this gap by assessing nitrogenous pollution from organic solvents and its effect on the nitrogen isotopic composition of nitrogen‐poor samples.  Firstly, Part I investigates potential nitrogenous pollution in combusted quartz powder introduced by commonly used laboratory solvents. The observed changes in TN and *δ* ^15^N in quartz powder solely reflect solvent‐derived pollution. Then, Part II further evaluates the accuracy of nitrogen concentration and isotope measurements on chert samples that naturally contain low nitrogen organic contents. Cherts with varying kerogen concentrations were tested with the least polluting cleaning protocol, such that the observed changes in TN and *δ* ^15^N in chert samples reflect a combination of potential addition of solvent‐derived pollution and removal of modern contamination during pre‐treatment.

## 2 Experimental

### 2.1 Materials

1. *Chemicals*. Organic solvents are widely used for removing modern contaminants in studies analyzing organic content and isotopic composition. In this study, three common solvents with varying polarity were selected: *n*‐hexane (95%, Optima, CAS 110–5403) and dichloromethane (DCM, Baker Analyzed and OmniSolv [Supporting Information], CAS 75‐09‐2) as non‐polar solvents, and ethanol (95%, VWR, MFN 89370‐082) as a polar solvent.
2. *Procedural controls*. In Part I, quartz powder was selected as the procedural controls for testing solvent‐hosted pollution because it can undergo identical sample handling steps to rock samples (e.g., powdering and pre‐treatment) and has negligible organic‐bound and silicate‐bound nitrogen contents. Although its low hydrocarbon absorption capacity compared to rock matrices may lead to an underestimation of contamination [7], its mineralogical composition closely matches that of our chert samples, which are composed of microcrystalline quartz. Therefore, quartz powder serves as an appropriate mineralogical proxy for procedural controls. To remove any indigenous or residual organic matter, the quartz powder was baked under an oxygen‐rich atmosphere at 500°C overnight prior to pre‐treatment.
3. *Samples*. In Part II, three chert samples, each with varying kerogen contents, were selected for analysis. Paleoarchean cherts, including our samples from the Dresser formation (81 031) and the Strelley Pool formation (PC03‐032 and PC04‐006), contain indigenous traces of organic materials, and their extracted kerogen has been previously analyzed for nitrogen concentration and isotopic composition using step‐combustion analysis [13, 14, 15].

### 2.2 Experimental Plan

#### 2.2.1 Part I: Investigating Potential Nitrogenous Pollution by Laboratory Reagents on Procedural Controls

In this part, we evaluated the effects on nitrogen concentration and isotopic values of pre‐treated procedural controls to assess potential pollution introduced by commonly used laboratory solvents for decontamination. The procedural control is quartz powder, which was baked at 500°C overnight to remove residual organic materials, allowing them to serve as blanks to monitor solvent‐derived pollution during pre‐treatment. Five pre‐treatment protocols were tested: (1) DCM‐only, (2) DCM + ethanol, (3) *n‐*hexane‐only, (4) *n*‐hexane + ethanol, and (5) ethanol only. Procedural controls were prepared and pre‐treated with these different laboratory reagents as described in Section 2.3.1 and subsequently analyzed for nitrogen concentration and isotopic composition. The pre‐treatment protocol with the least impact on both parameters was selected for application in low nitrogen content chert samples in the subsequent experiment (Part II).

#### 2.2.2 Part II: Evaluating Potential Nitrogenous Pollution by Laboratory Reagents on Low‐Nitrogen‐Content Chert Samples

In this part, three chert samples with varying organic contents were selected for pre‐treatment using the laboratory solvent identified in Part I as introducing the least nitrogenous pollution. Paleoarchean cherts contain indigenous traces of organic materials. To assess solvent‐derived pollution and modern contamination removal in natural rock samples with low nitrogen contents, these cherts were not baked prior to pre‐treatment. The chert samples were processed as outlined in Section 2.3.1 and subsequently analyzed for nitrogen concentration and isotope composition, as described below.

### 2.3 Protocols

#### 2.3.1 Sample Preparation and Pre‐Treatment

Chert samples were cut into centimeter‐sized chips, and the outer surfaces were polished off with carborundum grit. Both the chips and quartz sand were then pulverized using an aluminum oxide ceramic puck mill. The mill was cleaned between samples using sand that has been baked overnight at 500°C, followed by rinsing with deionized water and ethanol to minimize cross‐contamination. In the pre‐treatment protocol, solvents were applied to quartz powder and cherts to ensure the removal of modern organic pollution while maintaining consistent sample handling. Approximately 1 g of each sample was weighed into overnight‐baked glass tubes and soaked overnight in either *n*‐hexane or DCM; selected samples were subsequently rinsed with ethanol. Following solvent treatment, the powdered chert samples were de‐carbonated using 6‐N HCl at 60°C for 3 days, with daily acid refreshment and stirring. Residual acid was removed by triple rinsing with deionized water, and the samples were then dried at 60°C.

#### 2.3.2 Nitrogen Concentration and Nitrogen Isotope Analyses

All analyses were carried out at the University of Washington Isolab facilities. TN concentration and nitrogen isotope ratios (*δ* ^15^N) were determined by bulk analysis using a Eurovector Elemental Analyzer coupled to a Thermo Finnigan MAT253 continuous flow isotope ratio mass spectrometer, as previously described [16]. Approximately 500 mg of powdered sample was weighed into double tin (9 × 10 mm) capsules, which were then flash combusted at 1100°C under an oxygen‐rich atmosphere. Resulting N_2_ and CO_2_ gases were purified with chromium oxide at 1100°C to convert trace CO to CO_2_ and with silvered cobalt oxide to capture sulfur gases and halogens. Then another purification step with copper at 650°C converted trace NOx to N_2_. Traces of H_2_O were scrubbed with magnesium perchlorate. Subsequently, N_2_ and CO_2_ gases were separated chromatographically in a gas chromatography column and analyzed for their isotopic compositions. To ensure complete combustion, each analytical run was bracketed by a blank run; background nitrogen in the blanks was negligible and below detection limits.

Nitrogen isotope ratios are reported in delta notation (*δ* ^15^N, ‰) relative to atmospheric N_2_ (AIR, *δ* ^15^N = 0‰) according to *δ* ^15^N (‰) = [(*R*_sample_/*R*_Standard_) − 1] × 1000, where *R* = ^15^N/^14^N. Isotopic values were calibrated using a two‐point linear normalization against two in‐house standards: GA2 (glutamic acid, *δ* ^15^N = −5.7‰) and SA (dried salmon, *δ* ^15^N = +11.3‰), which were calibrated against the international reference materials USGS40 and USGS41 [17]. A third in‐house standard, GA1 (glutamic acid, *δ* ^15^N = −4.6‰), was treated as an independent standard to assess analytical measurement; the average accuracy (0.41‰, 1*σ*) and precision (0.21‰, 1*σ*) of *δ* ^15^N were calculated as the mean offset between the measured and accepted values of GA1 across all runs. Every three samples were bracketed by a set of three standards to monitor instrumental drift. Nitrogen concentration was determined by a calibration curve of GA1 at varying masses.

Analytical reproducibility was assessed using the mean absolute deviation (MAD) rather than standard deviation (SD), as MAD is less sensitive to outliers, which was an issue given the elevated variability observed in low‐nitrogen samples. Replication represented the largest source of analytical uncertainty and was used to represent the error of individual sample measurements. All samples were analyzed with at least three replicates, except where sample material was limited.

## 3 Results

### 3.1 Part I: Investigating Potential Nitrogenous Pollution by Laboratory Reagents on Procedural Controls

Procedural controls of powdered quartz were pre‐treated by different laboratory reagents to investigate potential nitrogenous pollution. Prior to pre‐treatment, powdered quartz was baked at 500°C overnight to establish a background baseline and showed a reduction in average TN concentration from 8.6 ± 0.2 ppm (*n = 3*) to 5.8 ± 0.7 ppm (*n = 4*). However, the average of *δ* ^15^N values showed no statistically significant difference, with overlapping ranges from 7.1‰ ± 0.4‰ to 7.3‰ ± 2.0‰.

**FIGURE 1:**
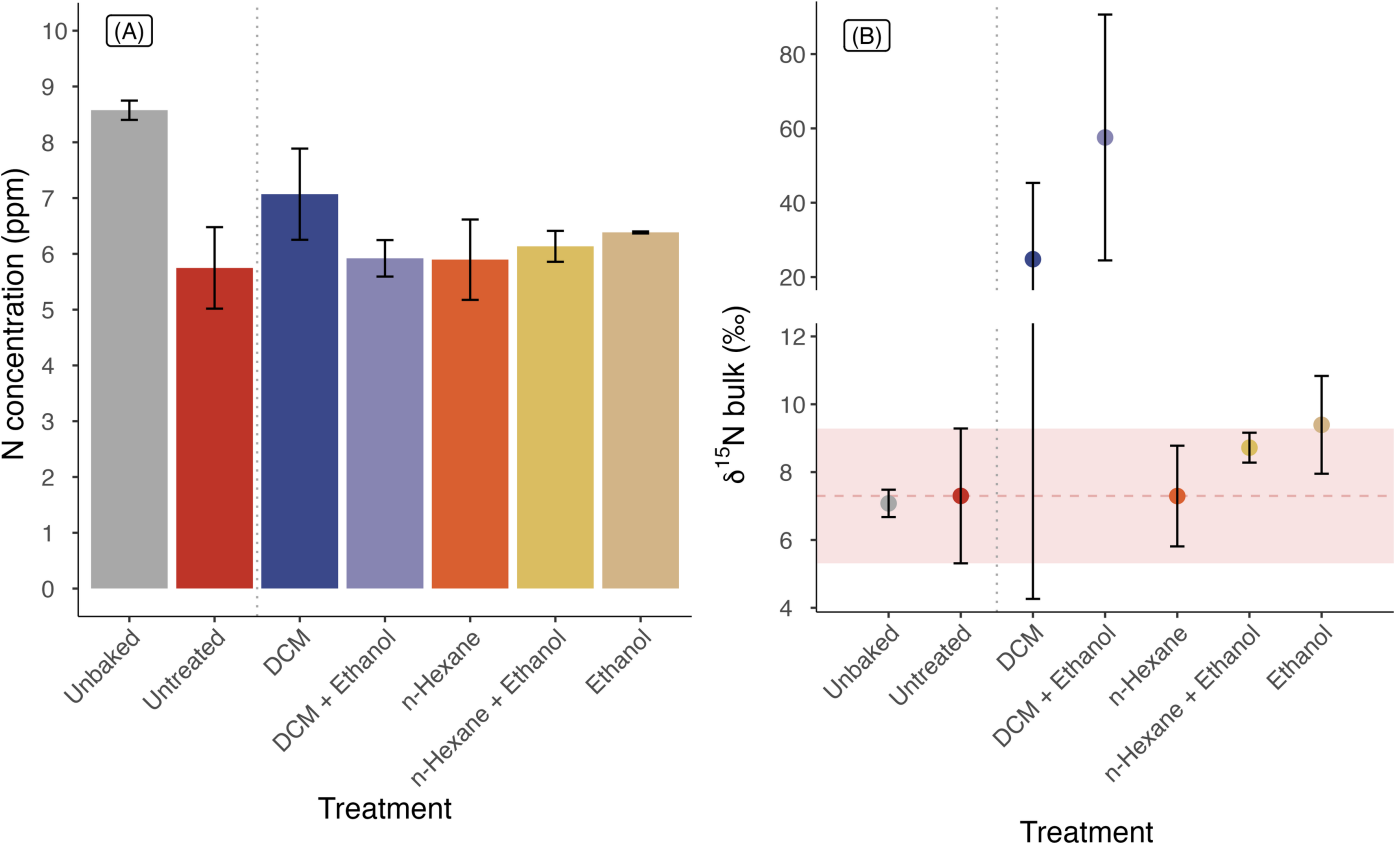
Total nitrogen concentration (A) and nitrogen isotopic composition (B) of procedural controls before and after different pre‐treatments; unbaked samples (*n* = 3), untreated samples (*n* = 4), DCM‐only (*n* = 4), DCM followed by ethanol (*n* = 4), *n*‐hexane‐only (*n* = 4), and *n*‐hexane followed by ethanol (*n* = 4) and ethanol only (*n* = 3). The red horizontal dashed line and red shaded area depict the background baseline of the average *δ* ^15^N of untreated quartz powder (7.3‰ ± 2.0‰). *Note:* For Panel (B) the scale of *y* axis breaks between *δ* ^15^N values of 12‰ to 20‰ for easier visualization. Error bars represent one median absolute deviation. Data are included in Table S1.

Then, the baked quartz samples, now referred to as untreated controls, underwent different pre‐treatments, with the TN and *δ* ^15^N values of different pre‐treated controls depicted in Figure 1 and listed in Table S1. All pre‐treatments showed slightly increased TN concentrations in pre‐treated controls compared to untreated controls (Figure 1A). The highest increases in TN concentration after pre‐treatments are listed in order: DCM (+1.3 ppm), ethanol (+0.6 ppm), *n*‐hexane followed by ethanol (+0.4 ppm), DCM followed by ethanol (+0.2 ppm), and *n*‐hexane (+0.2 ppm).

Although all pre‐treatments showed minimal increases in nitrogen contents (< 2 ppm), the nitrogen isotopic composition (*δ* ^15^N) for quartz powders varied between treatments over a range of 7.1‰ to 57.6‰ (with a median of 8.2‰) (Figure 1B and Table S1). A Kruskal–Wallis test indicated that there were significant differences between treatment groups (*p* = 0.024). However, post hoc pairwise comparisons did not identify any significant differences between individual groups (all *p* > 0.05). Given the limited statistical power due to small sample numbers and the exploratory nature of this study, we simply compared pre‐treatment means with untreated controls. The average *δ* ^15^N values of *n*‐hexane with ethanol pre‐treated controls (mean ±1 median absolute deviation: 8.7‰ ± 0.4‰, *n* = 4), the *n*‐hexane‐only pre‐treated controls (7.3‰ ± 1.5‰, *n* = 4), and the ethanol‐only pre‐treated controls (9.4‰ ± 1.4‰, *n* = 3) appeared similar to those of untreated controls (7.3‰ ± 2.0‰, *n* = 4), as indicated by the red dashed line and shaded area (Figure 1B). In contrast, the average *δ* ^15^N values of the DCM with ethanol pre‐treated controls (57.6‰ ± 33.1‰, *n* = 4) and the DCM‐only pre‐treated controls (24.8‰ ± 20.5‰, *n* = 4) were markedly distinct from those of untreated controls.

The *δ* ^15^N values from both *n*‐hexane‐only and *n*‐hexane with ethanol treatments were the most consistent with those of the untreated samples. Although the *n*‐hexane‐only pre‐treatment had the least effect on TN content and *δ* ^15^N values in untreated controls, modern organic contamination likely involves a wide range of substances. Therefore, double‐solvent pre‐treatment is recommended for more effective removal of both polar and non‐polar contaminants. Thus, the *n*‐hexane with ethanol pre‐treatment was selected for further testing on low TN‐cherts. The rationale is further detailed in Section 4.

### 3.2 Part II: Assessment of Pre‐Treatment on *δ* ^15^N Precision in Nitrogen‐Poor Cherts

**FIGURE 2:**
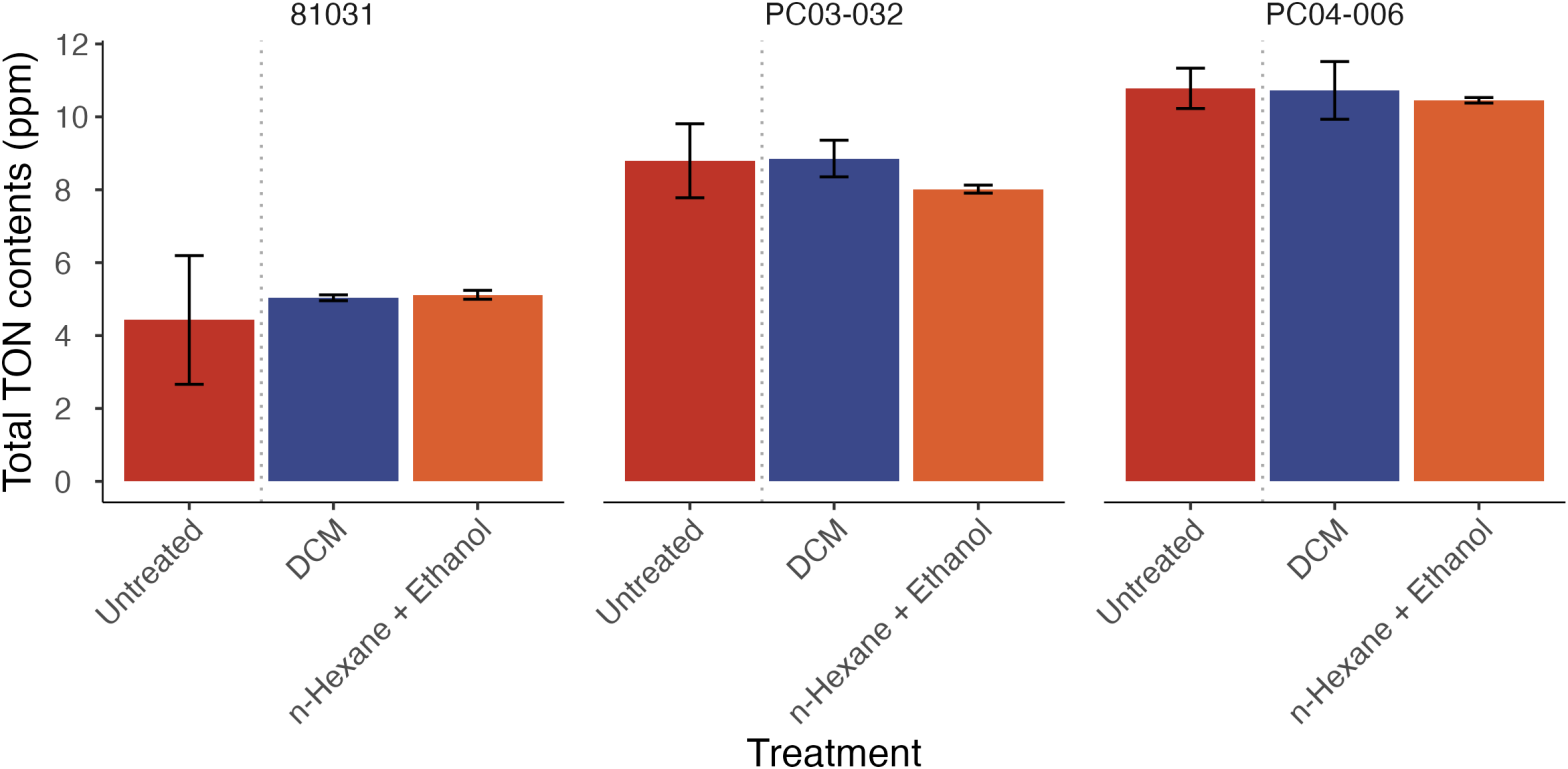
Comparison of total nitrogen (TN) concentration of untreated and pre‐treated chert samples with varying TN contents. Error bars represent one median absolute deviation. Data are included in Table S2.

The relationship of TN content and *δ* ^15^N values after pre‐treatments differed among low‐TN chert samples with varying TN contents: 81 031 (4.4 ± 1.8 ppm, *n* = 7), PC03‐032 (8.8 ± 1.0 ppm, *n* = 7), and PC04–006 (10.8 ± 0.6 ppm, *n* = 7), as shown in Figure 2 and listed in Table S2. After pre‐treatments, each sample showed minimal change in TN (either increase or decrease, < 1 ppm) and a small range in *δ* ^15^N values (4.0‰ to 8.4‰). Although trends were observed, the small sample numbers for each treatment (*n* = 3) may have limited the ability to detect statistically significant differences.

## 4 Discussion

### 4.1 The Background Baseline of TN and *δ* ^15^N Values of Procedural Controls

The comparison of nitrogen concentration and isotopic effects in untreated and pre‐treated procedural controls can assess the potential pollution introduced by commonly used laboratory solvents. To establish a background baseline of procedural controls, powdered quartz was baked prior to pre‐treatments. These untreated controls showed a reduction in average TN concentration from 8.6 ± 0.2 ppm (*n = 3*) to 5.8 ± 0.7 ppm (*n = 4*), suggesting that some organic contaminants were removed by baking at 500°C. This excess nitrogenous organic matter in unbaked samples could have been accumulated during long‐term storage and sample handling. While organic contaminants were likely completely removed during baking, background nitrogen residues remained in the untreated controls, which probably represent indigenous nitrogenous impurities embedded in the crystalline structure of the quartz. Thus, the TN and *δ* ^15^N of untreated controls (5.8 ± 0.7 ppm and *δ* ^15^N of 7.3‰ ± 2.0‰), as depicted by the red bar in Figure 1A and the red dashed line and shading in Figure 1B, serve as a baseline to compare with other controls that have undergone different pre‐treatments. Any observable changes in TN and *δ* ^15^N in pre‐treated controls thus record potential pollution from organic solvents.

### 4.2 Part I: Investigating Pollution by Laboratory Reagents on Procedural Controls

All pre‐treatments slightly increased the TN content in pre‐treated controls compared to untreated controls, suggesting nitrogen addition from the organic solvents themselves. To evaluate the extent of such pollution introduced by individual solvents, the TN contents in controls subjected to a single‐solvent pre‐treatment were compared. DCM solvent introduced the most nitrogenous pollution (+1.3 ppm), followed by ethanol (+0.6 ppm), and *n*‐hexane introduced the least amount (+0.1 ppm). This is further supported by evidence that DCM solvent extraction may retain some nitrogen‐containing compounds, leading to elevated nitrogen concentration in the recovered product [18, 19]. Moreover, the use of DCM during pre‐treatment significantly enriches organic carbon in the final product [12], consistent with our observations.

In addition, this study tested paired‐solvent pre‐treatments to evaluate their effectiveness and the extent of solvent‐associated pollution. The use of both polar (e.g., ethanol) and non‐polar (e.g., DCM or *n*‐hexane) solvents could be expected to increase the efficiency of contaminant removal by targeting a broad range of compounds with varying polarities. The *n*‐hexane + ethanol pre‐treatment increased TN content by 0.3 ppm, whereas the DCM + ethanol pre‐treatment increased TN content by 0.1 ppm. Although these changes are not statistically different, the TN content from paired‐solvent treatments was lower than the sum of their individual solvent contributions, suggesting that sequential pre‐treatment with a non‐polar solvent followed by a polar one may reduce overall pollution, such that the later ethanol step may help remove residues introduced by the initial non‐polar solvent (DCM or *n*‐hexane). Compared to single‐solvent pre‐treatment (DCM‐only or *n*‐hexane‐only), the paired‐solvent pre‐treatment (DCM + ethanol or n‐hexane + ethanol) added less than 0.5‐ppm nitrogen. Given that the principal goal of pre‐treatment is to remove all possible kinds of modern contamination, paired‐solvent pre‐treatment is favored. Although the use of multiple solvents may introduce more solvent‐derived pollution, the risk could be minimal. This risk was further assessed with *δ* ^15^N values from low TN samples in Part II.

Although all single‐solvent and pair‐solvent treatments showed minimal enrichment in nitrogen contents (< 2 ppm), the *δ* ^15^N for quartz powders varied significantly between pre‐treatments (Figure 1B), highlighting distinct isotopic effects and helping assessment of the extent of solvent‐derived pollution. Notably, pre‐treatments involving DCM resulted in a large deviation of *δ* ^15^N values from untreated controls. The DCM‐only pre‐treatment showed a broad isotopic range of +24.8 ± 20.5‰, while the DCM + ethanol pre‐treatment yielded dramatically positive *δ* ^15^N values of +57.6‰ ± 33.1‰. Both averages lie well outside the uncertainty range of the untreated controls, indicating clear isotopic perturbation. Pre‐treatment with a different commercial brand of DCM also yielded different values of *δ* ^15^N (Figure S1 and Table S3). DCM has a boiling point at 39.6°C, making it highly volatile at room temperature (During the soaking step, the volume decreased by half overnight). The large isotopic perturbation likely reflects preferential evaporation of a ^14^N‐enriched fraction or the isotopic values of retained nitrogenous compounds, though further experiments are needed to confirm this. Overall, the large isotope variation indicates that the signal is unlikely to be indigenous and instead reflects pollution from DCM.

Similarly, ethanol‐only pre‐treatment yielded *δ* ^15^N values (+9.4‰ ± 1.4‰) that also exceeded the untreated control range, confirming a measurable difference. In contrast, both *n*‐hexane pre‐treatments showed minimal deviation of *δ* ^15^N values and remained within the uncertainty range of untreated samples: the *n*‐hexane‐only pre‐treatment had a slightly lower mean *δ* ^15^N value (+7.3‰ ± 1.5‰), whereas the *n*‐hexane + ethanol pre‐treatment exhibited a slightly higher mean (+8.7‰ ± 0.4‰). Overall, both results are consistent with those of the untreated baseline.

Although the *n*‐hexane‐only pre‐treatment resulted in the least perturbation of TN contents and *δ* ^15^N values compared to untreated controls, as mentioned earlier, paired‐solvent pre‐treatment was preferred for targeting a broad range of pre‐treatment contaminant compounds. Thus, the *n*‐hexane with ethanol pre‐treatment was considered a reasonable compromise result and was then selected for Part II of the study.

### 4.3 Part II: Assessment of Pre‐Treatment Impacts on *δ* ^15^N Precision in Low‐TN Cherts

To assess the impact of solvent‐derived contamination on *δ* ^15^N in low TN geological samples, three Archean chert samples with varying TN contents were selected: 81 031 (4.4 ± 1.8 ppm, *n* = 7), PC03‐032 (8.8 ± 1.0 ppm, *n* = 7), and PC04‐006 (10.8 ± 0.6 ppm, *n* = 7). Each sample underwent pre‐treatment with *n*‐hexane + ethanol, the preferred protocol identified in Part I. In addition, DCM‐only pre‐treatment, which is commonly used in organic geochemical analysis, was included for comparison.

The *δ* ^15^N values of untreated chert samples did not accurately reflect the primary *δ* ^15^N signal because they contained post‐depositional contamination during storage and handling. Pre‐treatments could lead to the removal of modern nitrogenous contaminants as well as the addition of solvent‐derived nitrogen. In this study, the average TN contents and *δ* ^15^N for each pre‐treatment were compared to those of untreated samples to evaluate the relative effects of different solvents across samples with varying TN contents.

After pre‐treatments, each chert sample showed a narrow range in *δ* ^15^N values (+4.0‰ to +8.4‰), consistent with the broad range of *δ* ^15^N values reported for kerogen in similar cherts in previous studies (−6.2‰ to +13.0‰ [13, 14, 15, 20]). However, those studies did not perform bulk‐rock measurements, as was done here. Given that these cherts have undergone only low‐grade metamorphism, bulk analysis is a better method for nitrogen isotope analysis than kerogen extraction [21]. For this reason, the accuracy of the previously reported primary *δ* ^15^N values cannot be independently verified.

**FIGURE 3:**
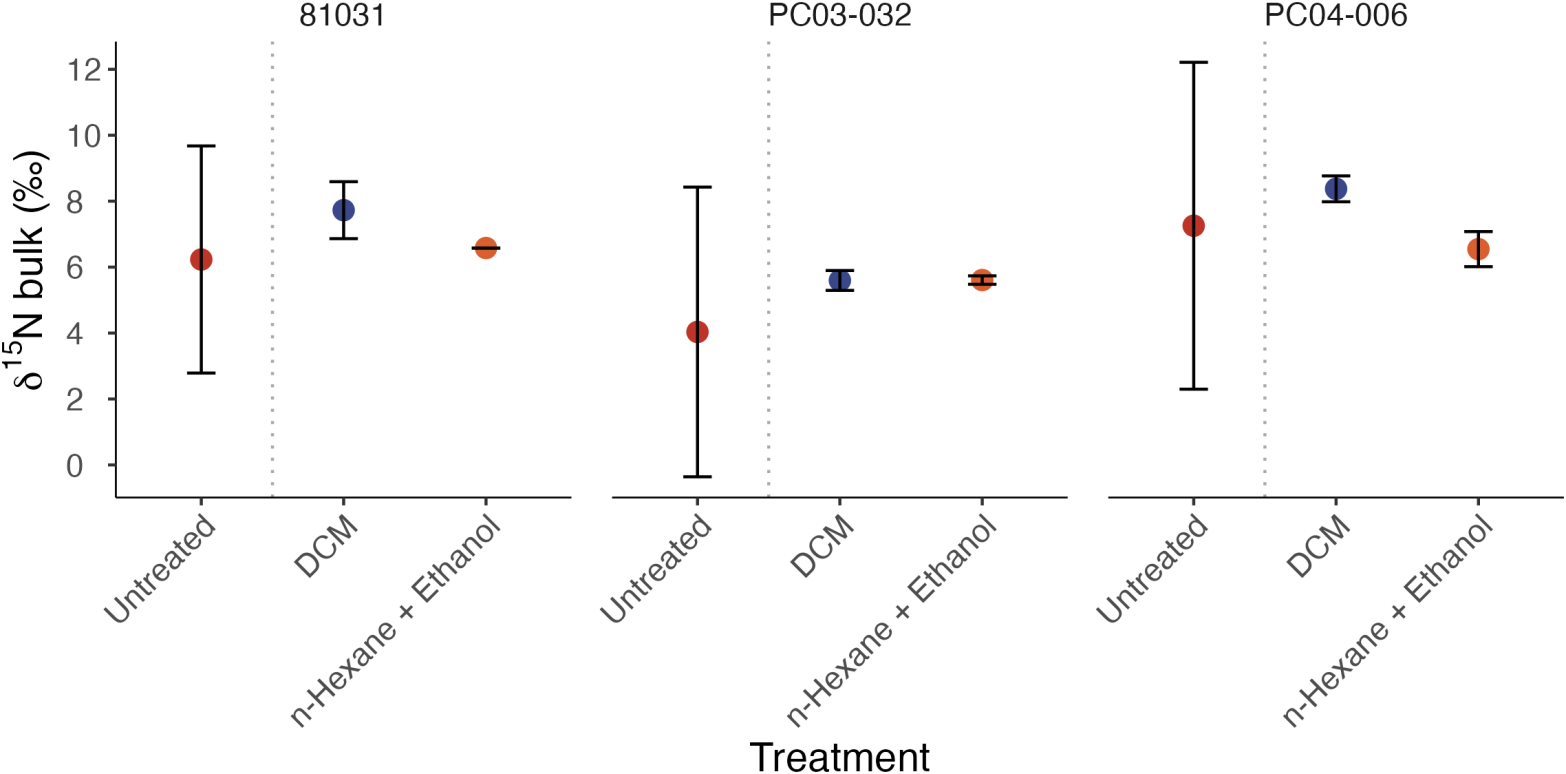
Comparison of *δ* ^15^N values of untreated and pre‐treated chert samples with varying TN contents. Error bars represent one median absolute deviation. Data are included in Table S2.

The relationship of TN and *δ* ^15^N after pre‐treatments differed between chert samples with varying TN contents, as shown in Figures 2 and 3. For DCM‐only treated cherts, most of them exhibited an increase in both TN and *δ* ^15^N values, likely reflecting a greater effect from DCM‐derived nitrogen addition rather than the removal of nitrogenous contaminants. The high‐TN sample (PC04‐006; > 10 ppm) exhibited only a minimal TN decrease (0.06 ppm), whereas the low‐TN samples (81 031 and PC03‐032; < 10 ppm) showed variable TN increases of up to 0.6 ppm. These results suggest that DCM‐derived nitrogen pollution is more pronounced in nitrogen‐poor samples and that DCM may be ineffective at removing modern organic contaminants. Moreover, all samples exhibited increased *δ* ^15^N values following DCM‐only treatment. These elevated *δ* ^15^N values suggest that DCM introduced nitrogen pollution with a distinct isotopic signature, as observed in the DCM‐treated control in Part I, which may potentially mask the true primary *δ* ^15^N variability between samples. Therefore, DCM‐only treatment is unreliable for removing modern contamination, as it introduces nitrogen pollution that may obscure the primary *δ* ^15^N values in low‐TN samples.

Most samples treated with *n*‐hexane + ethanol showed decreased TN and varying *δ* ^15^N values, likely reflecting more effective removal of modern nitrogenous contaminants without the addition of much nitrogenous pollution from solvents. While the lowest‐TN sample (81 031; < 8 ppm) exhibited a TN increase of 0.7 ppm, this likely reflects minor solvent‐derived nitrogen pollution, supported by a slight enrichment in ^15^N values as observed in Part I. In contrast, higher‐TN samples (PC03‐032 and PC04‐006; > 8 ppm) showed TN decreases of 0.8 and 0.3 ppm, respectively, indicating that contaminant removal outweighed solvent addition in these samples. Moreover, these two samples exhibited opposing *δ* ^15^N trends: PC03‐032 showed ^15^N enrichment, while PC04‐006 showed depletion. This likely reflects the removal of compositionally distinct contaminants, resulting in the different *δ* ^15^N shift. In PC03‐032, the smaller *δ* ^15^N deviation suggests that pre‐treatment effectively removed modern contamination and those *δ* ^15^N values likely reflect the primary *δ* ^15^N signal. While PC04–006 samples exhibited high *δ* ^15^N variability in both untreated and pre‐treated samples, pre‐treatment reduced the *δ* ^15^N variation which suggests that the *n*‐hexane–ethanol pre‐treatment was effective. The remaining large variation may stem from inherent heterogeneity in the primary *δ* ^15^N composition. Due to limited sample material, additional measurements to further constrain uncertainty were not possible. Overall, these results suggest that *n*‐hexane + ethanol pre‐treatment can effectively remove modern nitrogen contamination in samples with TN as low as 9 ppm without significant isotopic pollution by solvent‐derived nitrogen.

## 5 Conclusion

Nitrogen isotope values have been widely used for tracing biogeochemical processes on the early Earth, but the effects of commonly used laboratory chemicals for removing more modern contaminants on the nitrogen contents and isotopic compositions of such ancient materials remain unquantified. This study evaluated the potential for nitrogen pollution from the laboratory solvents dichloromethane (DCM), *n*‐hexane, and ethanol during pre‐treatment of samples intended to remove modern organic contaminants introduced through post‐depositional geological processes and in various stages of sample collection and preparation. Among the single‐solvent treatments, *n*‐hexane introduced the least nitrogen pollution compared to ethanol and DCM. Despite this, we recommend the use of both polar and non‐polar solvents together to target the broadest range of organic contaminants with differing solubilities. Specifically, sequential pre‐treatment using *n*‐hexane followed by ethanol produced the most favorable outcome: it minimized solvent‐derived nitrogen addition while effectively removing modern contamination. Based on these findings, we recommend the paired‐solvent pre‐treatment of *n*‐hexane followed by ethanol for nitrogen isotope analysis, particularly in ancient low‐TN samples (as low as 9 ppm). This approach provides a practical compromise—effectively removing modern organic contamination without introducing significant reagent‐derived nitrogen while yielding more homogeneous *δ* ^15^N values. Moreover, given that DCM poses environmental and health hazards, its use should be minimized where possible in favor of safer and more effective alternatives.

## Author Contributions

**Roger Buick:** writing – review and editing, conceptualization, supervision, project administration, resources, funding acquisition. **Kunmanee Bubphamanee:** data curation, visualization, writing – review and editing, methodology, conceptualization, investigation, writing – original draft, project administration. **Andrew Schauer:** methodology, data curation, resources, writing – review and editing, investigation.

## Funding

This work was supported by the University of Washington. Open access publication was supported by the open publishing agreement between the University of Washington Libraries and Wiley.

## Supporting information


**Table S1:** The nitrogen concentration and nitrogen isotopic composition of all samples plotted in Part I: Investigating potential nitrogenous pollution by laboratory reagents on procedural controls.
**Table S2:** The nitrogen concentration and nitrogen isotopic composition of all samples plotted in Part II: Evaluating potential nitrogenous pollution by laboratory reagents on low‐nitrogen‐content chert samples.
**Figure S1:** Comparison of δ15N values of solvent‐derived pollution on procedural controls. Data are included in the table below (Table S3).
**Table S3:** The nitrogen concentration and nitrogen isotopic composition additional quartz samples. However, these data were not included in the original plots due to the unparallel sample preparation, such that it cannot be compared directly.

## Data Availability

The data that support the findings of this study are available in the Supporting Information of this article.
