# Supplementary material for: Effect of Laboratory Reagents for Cleaning Modern Contamination on the δ 15N Integrity of Nitrogen‐Poor Samples
Source: Rapid Commun Mass Spectrom. 2026 Jul 16;40(19):e70136. doi: 10.1002/rcm.70136 (PMC13374597; doi:10.1002/rcm.70136)
Supplement: Supplementary file 1 — Table S1: The nitrogen concentration and nitrogen isotopic composition of all samples plotted in Part I: Investigating potential nitrogenous pollution by laboratory reagents on procedural controls. Table S2: The nitrogen concentration and nitrogen isotopic composition of all samples plotted in Part II: Evaluating potential nitrogenous pollution by laboratory reagents on low‐nitrogen‐content chert samples. Figure S1: Comparison of δ15N values of solvent‐derived pollution on procedural controls. Data are included in the table below (Table S3). Table S3: The nitrogen concentration and nitrogen isotopic composition additional quartz samples. However, these data were not included in the original plots due to the unparallel sample preparation, such that it cannot be compared directly. [file RCM-40-e70136-s001.docx]

**Supplementary Information**

**Table S1** The nitrogen concentration and nitrogen isotopic composition of all samples plotted in Part I: Investigating potential nitrogenous pollution by laboratory reagents on procedural controls.

| **Sample** | **Condition** | **δ^15^N (‰)** | **TN (ppm)** |
| --- | --- | --- | --- |
| Quartz powder | Unbaked | 6.19 | 8.46 |
| Quartz powder | Unbaked | 7.66 | 8.91 |
| Quartz powder | Unbaked | 7.39 | 8.35 |
| Average (n = 3) | | 7.1 ± 0.4 | 8.57 ± 0.17 |
| Quartz powder | Untreated | 4.86 | 6.28 |
| Quartz powder | Untreated | 10.36 | 6.22 |
| Quartz powder | Untreated | 6.44 | 5.30 |
| Quartz powder | Untreated | 7.54 | 5.19 |
| Average (n = 4) | | 7.3 ± 2.0 | 5.75 ± 0.73 |
| Quartz powder | DCM | 7.40 | 6.87 |
| Quartz powder | DCM | 40.87 | 8.63 |
| Quartz powder | DCM | 13.17 | 6.94 |
| Quartz powder | DCM | 37.75 | 5.84 |
| Average (n = 4) | | 24.8 ± 20.5 | 7.1 ± 0.8 |
| Quartz powder | DCM + Ethanol | 12.06 | 5.36 |
| Quartz powder | DCM + Ethanol | 149.11 | 5.41 |
| Quartz powder | DCM + Ethanol | 56.68 | 5.80 |
| Quartz powder | DCM + Ethanol | 12.36 | 7.11 |
| Average (n = 4) | | 57.6 ± 33.1 | 5.9 ± 0.3 |
| Quartz powder | n-Hexane | 11.00 | 5.40 |
| Quartz powder | n-Hexane | 6.77 | 5.29 |
| Quartz powder | n-Hexane | 4.76 | 6.27 |
| Quartz powder | n-Hexane | 6.65 | 6.62 |
| Average (n = 4) | | 7.3 ± 1.5 | 5.9 ± 0.7 |
| Quartz powder | n-Hexane + Ethanol | 7.83 | 6.65 |
| Quartz powder | n-Hexane + Ethanol | 7.95 | 6.66 |
| Quartz powder | n-Hexane + Ethanol | 10.68 | 6.29 |
| Quartz powder | n-Hexane + Ethanol | 8.42 | 4.94 |
| Average (n = 4) | | 8.7 ± 0.4 | 6.1 ± 0.3 |
| Quartz powder | Ethanol | 11.60 | 8.87 |
| Quartz powder | Ethanol | 10.63 | 5.15 |
| Quartz powder | Ethanol | 5.94 | 5.13 |
| Average (n = 3) | | 9.4 ± 1.4 | 6.4 ± 0.0 |

**Table S2** The nitrogen concentration and nitrogen isotopic composition of all samples plotted in Part II: Evaluating potential nitrogenous pollution by laboratory reagents on low-nitrogen-content chert samples.

| **Sample** | **Condition** | **δ^15^N (‰)** | **TN (ppm)** |
| --- | --- | --- | --- |
| 81031 | Untreated | 2.90 | 3.10 |
| 81031 | Untreated | 5.23 | 1.95 |
| 81031 | Untreated | 4.94 | 4.54 |
| 81031 | Untreated | 4.84 | 3.99 |
| 81031 | Untreated | 8.02 | 5.73 |
| 81031 | Untreated | 7.89 | 6.03 |
| 81031 | Untreated | 9.80 | 5.67 |
| Average (n = 7) | | 6.2 ± 3.4 | 4.4 ± 1.8 |
| 81031 | DCM | 6.53 | 4.83 |
| 81031 | DCM | 10.71 | 5.17 |
| 81031 | DCM | 5.95 | 5.11 |
| Average (n = 3) | | 7.7 ± 0.9 | 5.0 ± 0.1 |
| 81031 | n-Hexane + Ethanol | 6.60 | 5.00 |
| 81031 | n-Hexane + Ethanol | 6.60 | 5.27 |
| 81031 | n-Hexane + Ethanol | 6.53 | 5.09 |
| Average (n = 3) | | 6.6 ± 0.0 | 5.1 ± 0.1 |
| PC03-032 | Untreated | 6.96 | 9.09 |
| PC03-032 | Untreated | 6.92 | 9.13 |
| PC03-032 | Untreated | 6.75 | 9.06 |
| PC03-032 | Untreated | 3.38 | 8.40 |
| PC03-032 | Untreated | 3.96 | 5.17 |
| PC03-032 | Untreated | −0.26 | 10.36 |
| PC03-032 | Untreated | 0.52 | 10.36 |
| Average (n = 7) | | 4.0 ± 4.4 | 8.8 ± 1.0 |
| PC03-032 | DCM | 5.41 | 9.80 |
| PC03-032 | DCM | 6.16 | 8.21 |
| PC03-032 | DCM | 5.21 | 8.55 |
| Average (n = 3) | | 5.60 ± 0.3 | 8.9 ± 0.5 |
| PC03-032 | n-Hexane + Ethanol | 5.53 | 8.33 |
| PC03-032 | n-Hexane + Ethanol | 5.86 | 7.90 |
| PC03-032 | n-Hexane + Ethanol | 5.44 | 7.83 |
| Average (n = 3) | | 5.6 ± 0.1 | 8.0 ± 0.1 |
| PC04-006 | Untreated | 23.37 | 10.65 |
| PC04-006 | Untreated | 7.19 | 10.92 |
| PC04-006 | Untreated | 8.57 | 10.55 |
| PC04-006 | Untreated | 4.36 | 14.60 |
| PC04-006 | Untreated | 5.23 | 6.33 |
| PC04-006 | Untreated | 0.99 | 11.03 |
| PC04-006 | Untreated | 1.08 | 11.39 |
| Average (n = 7) | | 7.3 ± 5.0 | 10.8 ± 0.6 |
| PC04-006 | DCM | 7.72 | 10.05 |
| PC04-006 | DCM | 9.42 | 10.80 |
| PC04-006 | DCM | 7.99 | 11.33 |
| Average (n = 3) | | 8.4 ± 0.4 | 10.7 ± 0.8 |
| PC04-006 | n-Hexane + Ethanol | 4.54 | 10.60 |
| PC04-006 | n-Hexane + Ethanol | 7.37 | 10.21 |
| PC04-006 | n-Hexane + Ethanol | 7.73 | 10.55 |
| Average (n = 3) | | 6.6 ± 0.5 | 10.5 ± 0.1 |

**Commercial DCM causes variation in δ^15^N values in procedural controls**

In this study, procedural controls (quartz powder) were pre-treated with dichloromethane (DCM), a common organic solvent used during the organic extraction step. We conducted experiment with different commercial brands of DCM and found that they produce variation in δ^15^N values.

In this study, quart powder pre-treated with the first brand of DCM (label here as ‘DCM’, Dichloromethane, Baker Analyzed®, CAS 75-09-2) shows a wide range of nitrogen isotopic values, while the second brand (label here as ‘DCM2’, Dichloromethane, OmniSolv, Lot 51287, CAS 75-09-2) showed nitrogen isotopic composition of 1.5 ± 0.6‰.


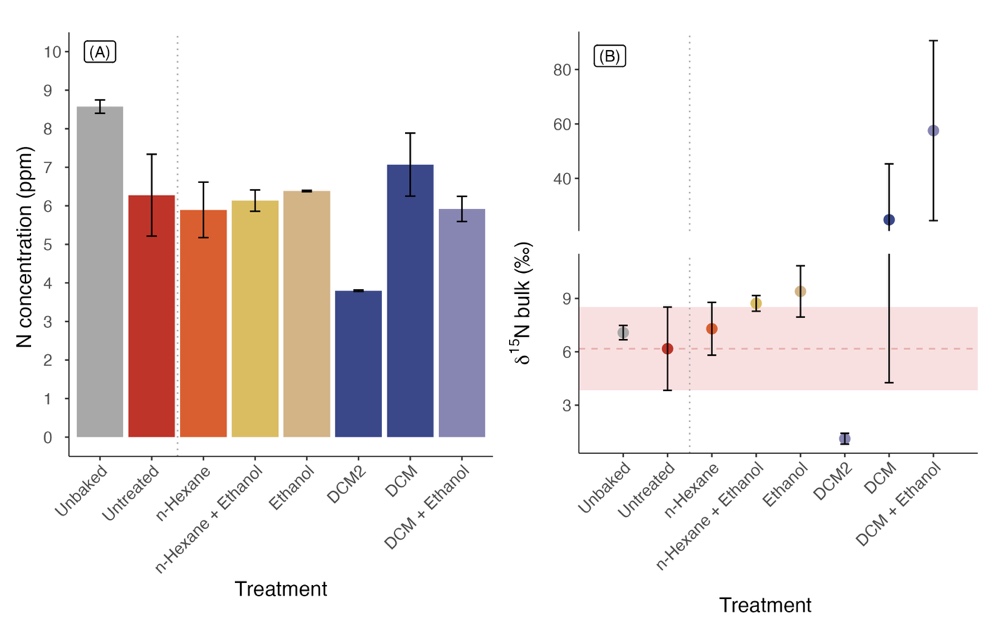


**Figure S1**: **Comparison of δ^15^N values of solvent-derived pollution on procedural controls.** Data are included in the table below (Table S3).

**Table S3** The nitrogen concentration and nitrogen isotopic composition additional quartz samples. However, these data were not included in the original plots due to the unparallel sample preparation, such that it cannot be compared directly.

| **Sample** | **Condition** | **δ^15^N (‰)** | **TN (ppm)** |
| --- | --- | --- | --- |
| Quartz powder | Untreated | 2.81 | 7.00 |
| Quartz powder | Untreated | 4.51 | 7.06 |
| Quartz powder | Untreated | 6.70 | 6.88 |
| Average (n = 3) | | 4.7 ± 1.9 | 7.0 ± 0.1 |
| Quartz powder | DCM2 | 1.59 | 3.83 |
| Quartz powder | DCM2 | 0.98 | 3.84 |
| Quartz powder | DCM2 | 0.77 | 3.72 |
| Quartz powder | DCM2 | 2.53 | 3.71 |
| Average (n = 4) | | 1.5 ± 0.6 | 3.8 ± 0.1 |

Previous work has also examined solvent-derived pollution on carbon contents and carbon isotopic composition, using quartz powder pre-treated with different DCM brands (Muller et a., 2016). Their results showed that DCM introduced the highest carbon contamination, with a mean carbon isotopic composition of −27.9‰, although exact values for each solution were not reported. Although we did not measure the carbon isotopic composition of our first DCM bottle and pre-treated samples, we measured those values in the additional study and found that pre-treated samples with the second brand DCM (label here as ‘DCM2’) had a carbon isotopic composition of −12.2 ± 1.5‰ (n=3). In addition, we suspected volatilization may influence the δ^13^C values, we performed additional test by allowing pre-treated samples to dryness at room temperature, instead of in the oven (60˚C), the resulting samples exhibited a much more negative values of a carbon isotopic composition of −24.5‰ (n=1) with approximately double amount of carbon contents.

As a result, the available carbon isotopic compositions of pre-treated samples differ from the previous study, suggesting the carbon isotopic compositions of DCM vary among commercial brands. That suggest the production process or substrate used in manufacturing DCM may vary among commercial brands. Thus, these findings support that DCM has the potential to introduce variability in δ^15^N values procedural controls.
